# The ever-expanding conundrum of primary osteoporosis: aetiopathogenesis, diagnosis, and treatment

**DOI:** 10.1186/1824-7288-40-55

**Published:** 2014-06-07

**Authors:** Stefano Stagi, Loredana Cavalli, Salvatore Seminara, Maurizio de Martino, Maria Luisa Brandi

**Affiliations:** 1 Health Sciences Department, University of Florence, Anna Meyer Children’s University Hospital, Florence, Italy; 2 Department of Internal Medicine, Endocrinology Unit, University of Florence, Florence, Italy

**Keywords:** Bone mineral density, Children, Fragility fractures, Osteopoenia, Osteoporosis, Primary osteoporosis

## Abstract

In recent years, as knowledge regarding the etiopathogenetic mechanisms of bone involvement characterizing many diseases has increased and diagnostic techniques evaluating bone health have progressively improved, the problem of low bone mass/quality in children and adolescents has attracted more and more attention, and the body evidence that there are groups of children who may be at risk of osteoporosis has grown. This interest is linked to an increased understanding that a higher peak bone mass (PBM) may be one of the most important determinants affecting the age of onset of osteoporosis in adulthood. This review provides an updated picture of bone pathophysiology and characteristics in children and adolescents with paediatric osteoporosis, taking into account the major causes of primary osteoporosis (PO) and evaluating the major aspects of bone densitometry in these patients. Finally, some options for the treatment of PO will be briefly discussed.

## Introduction

Recently, as knowledge regarding the aetiopathogenetic mechanisms of many diseases affecting bone health has increased and the diagnostic techniques for evaluating bone status have improved, the problem of low bone mass/quality in children and adolescents has received increased attention, revealing evidence of groups of children who may be at risk of osteoporosis [1,2]. This interest is linked to an increased understanding that a higher peak bone mass (PBM) may be one of the most important determinants affecting the age of onset of osteoporosis in adulthood [3,4]. Early diagnosis and therapeutic intervention are fundamental for ensuring better bone health in adulthood. For example, data gathered from 22q11 Deletion Syndrome patients suggests that a reduction in bone accretion could lead to a pathologically reduced bone mass in young adults [5]. Alternatively, many studies have shown that specific types of physical exercise undertaken during childhood and adolescence may determine bone health in adulthood, most likely by increasing PBM [6-8]. Thus, the achievement of an optimal PBM includes measures to block reductions in bone apposition or to increase bone remodelling and bone accrual.

Primary osteoporosis in children occurs due to an intrinsic bone abnormality (Primary Osteoporosis, PO) that is usually genetic in origin, whereas secondary osteoporosis arises from an underlying medical condition or its treatment [9]. In these forms, osteoporosis could be related to 1) failure to achieve optimal PBM in adolescence, 2) excessive resorption of bone, or 3) failure to adequately replace the resorbed bone through a bone formation deficit. Although PO is defined by decreased bone strength that predisposes individuals to fragility fractures, we have poor long-term follow-up data on bone health in many of the PO disorders, as well as on bone characteristics, growth, quality, and density in the pubertal or post-pubertal periods, on PBM, and on the effects of nutritional interventions or pharmacological treatments. In order to understand these issues, it is necessary to know the specific anatomical and physiological bone characteristics of children, in addition to the peculiarities in the diagnostic evaluation of bone density or quality. Because inadequate acquisition of bone in childhood and adolescence may lead to an increased lifetime risk of osteoporosis and fracture, it is important to recognise whether bone mineral status is affected in children with PO.

This review provides an updated picture of the pathophysiological basis of the main forms of PO in children and adolescents, taking into account the most common among the rare syndromes causing PO and evaluating the main aspects of bone densitometry in these patients. Finally, treatment options for the various forms of PO are briefly discussed.

### Primary osteoporosis

The primary forms of osteoporosis in childhood are relatively rare, and some of them are familial or genetically determined (Table 1). The most common primary bone disorder leading to PO is osteogenesis imperfecta (OI), a structural genetic defect in the quantity or quality of bone type 1 collagen production [10]. OI has several subtypes, ranging from mild forms to a form that causes intrauterine foetal death. Family history, the blue, purple, or grey sclera commonly observed in this disorder, radiographic findings, and in some cases, bone biopsy, are used to establish the diagnosis of the genetic disorder.

**Table 1:** Causes of primacy osteoporosis in children and adolescents

**Diseases**	**Genes**
Osteogenesis imperfecta	*COL1A1; COL1A2; IFITM5; SERPINF1; CRTAP; LEPRE1; PPIB; FKBP10; BMP1; SP7; SERPINH1; WNT1; TMEM38B*
X-linked hypophoshatemic rickets	*PHEX*
Homocystinuria	*CBS*
Hypophosphatasia	*ALPL*
Wilson’s disease	*ATP7B*
Menkes’ kinky hair syndrome	*ATP7A*
Osteoporosis-pseudoglioma syndrome	*LRP5*
Idiopathic juvenile osteoporosis	-
Juvenile Paget’s disease	*OPG*
Early-onset Paget’s disease	*RANK*
Ehler–Danlos syndrome	*COL5A2; COL5A1; COL1A1; COL3A1; PLOD1; COL1A2; ADAMTS2; COL3A1; TNXB*
Bruck syndrome	*FKBP10; PLOD2*
Marfan syndrome	*FBN1*
Hypophosphatemic nephrolithiasis/osteoporosis	*SLC34A1; NPHLOP2*
Hajdu-Cheney syndrome	*NOTCH2*
Torg-Winchester syndrome	*MMP2*
Shwachman-Diamond syndrome	*SBDS*
Singleton-Merten syndrome	-
Cleidocranial dysostosis	*RUNX2*
Stuve-Wiedemann syndrome	*LIFR*
Cole-Carpenter syndrome	-
Geroderma osteodysplasticum	*GORAB*
Noonan syndrome	*PTPN11; SHOC2; KRAS*; *SOS1; RAF1; NRAS; BRAF; RIT1*
Neonatal hyperparathyroidism	*CASR*
Other forms of hypophosphatemic rickets	*SLC34A3; FGF23; DMP1; ENPP1; CLCN5*
Hypocalcemic rickets	*VDR; CYP2R1; CYP27B1*

Note. This table lists only the most frequent diseases associated with primary osteoporosis according to the recent literature.

Other causes of PO include idiopathic juvenile osteoporosis (IJO), osteoporosis-pseudoglioma syndrome (OPPG), and X-linked hypophosphatemic rickets (XLH) [11]. Enzymatic defects such as hypophosphatasia and homocystinuria as well as disorders of copper transport such as Wilson’s disease and Menkes’ kinky hair syndrome can also lead to osteoporosis or severe demineralisation [12,13]. Finally, some genetic syndromes, such as Marfan and Ehler–Danlos syndrome, or more rare genetic diseases such as Hajdu-Cheney, Torg-Winchester and Shwachman-Diamond syndromes, are associated with an impairment of bone mass or quality.

#### ***Osteogenesis imperfecta***

OI, or brittle bone disease, a rare heritable connective tissue disorder, is classified as a form of osteoporosis, even if the primary defect is genetically altered structure of type I collagen composing the bone matrix [14]. Typical features of OI are multiple peripheral and vertebral compression fractures, blue sclera, excessive joint laxity, dentinogenesis imperfecta, and hearing loss [14]. The original classification by Sillence based on phenotypic features consisted of four types that vary in severity [15]. OI has a birth prevalence of approximately 6 to 7 in 100,000 [16], differing by type, with OI type I and OI type IV accounting for more than half of all OI cases [16]. OI type I has a prevalence of 3 to 4 per 100,000 and an incidence of 3.5 per 100,000, whereas the incidence of OI type II is approximately 1 to 2 per 100,000 (the prevalence is not available due to early lethality) [15,16]. Finally, OI type III has an incidence of 1.6 per 100,000 and a prevalence of 1 to 2 per 100,000 [15,16]. OI type IV, which is similar to the other forms, is believed to be rare.

OI is usually characterised by autosomal dominant inheritance (95% of cases), but some cases are related to autosomal recessive traits or to a spontaneous mutation [15,17] (Table 2). The OI type I (*OMIM #166200*) phenotype can be produced by a mutation in either *COL1A1* (*OMIM +120150*) or *COL1A2* (OMIM *120160), and possibly in other genes. Type II OI (*OMIM #166210*) is lethal in the perinatal period. Type III (*OMIM #259420*) is a severe form with obvious bony deformities and reduced BMD [17]. Whereas type I OI patients have a reduced amount of type I collagen, patients with types II, III and IV (*OMIM #166220*) have lower quality type I collagen [9,18]. Some children with OI do not fall clearly into one of these four types. In recent years, other additional forms of OI have been identified (types V-XV) based on a combination of phenotypic and bone histological features [19,20]. For OI classification, we have used the nomenclature reported in the Online Mendelian Inheritance in Man (OMIM; http://www.ncbi.nlm.nih.gov/omim) (Table 2).

**Table 2:** Causes of osteogenesis imperfecta, involved genes, location, inheritance, and gene products

**Osteogenesis imperfecta**	**OMIM**	**Inheritance**	**Gene**	**Location**	**Gene product**
Type I	166200	AD	*COL1A1*	17q21.33	Collagen, Type I, Alpha-1
Type II	166210	AD	*COL1A1*	17q21.33	Collagen, Type I, Alpha-1
	166210	AD	*COL1A2*	7q21.3	Collagen, Type I, Alpha-2
Type III	259420	AD	*COL1A1*	17q21.33	Collagen, Type I, Alpha-1
	259420	AD	*COL1A2*	7q21.3	Collagen, Type I, Alpha-2
Type IV	166220	AD	*COL1A2*	7q21.3	Collagen, Type I, Alpha-2
	166220	AD	*COL1A1*	17q21.33	Collagen, Type I, Alpha-1
Type V	610967	AD	*IFITM5*	11p15.5	Interferon-induced transmembrane protein-5
Type VI	613982	AR	*SERPINF1*	17p13.3	Serpin peptidase inhibitor
	610682	AR	*CRTAP*	3p22.3	Cartilage-associated protein
	610915	AR	*LEPRE1*	1P34.2	Leucine- and Proline-Enriched Proteoglycan 1
Type IX	259440	AR	*PPIP*	15q22.31	Peptidyl-prolyl isomerase b
Type X	613848	AR	*SERPINH1*	11q13.5	Serpin peptidase inhibitor, Clade H, Member 1
Type XI	610968	AR	*FKBP10*	17q21.2	FK506-binding protein 10
Type XII	613849	AR	*SP7*	12q13.13	Transcription factor Sp7
Type XIII	614856	AR	*BMP1*	8q21.3	Bone morphogenetic protein 1
Type XIV	615066	AR	*TMEN38B*	9q31.2	Transmembrane Protein 38B
Type XV	615220	AR	*WNT1*	12q13.12	Wingless-type MMTV Integration Site Family, Member 1

Note. This table lists only the most frequent types according to the recent literature.

In OI, musculoskeletal abnormalities include long bone deformities, with anterior bowing of the humerus, tibia and fibula, and lateral bowing of the femur, radius and ulna [9]. Patients have varying degrees of skeletal fragility, with fractures and bone deformities often occurring after trivial trauma and manifesting independently of clinical severity [14]. Other clinical OI manifestations include short stature, blue sclera, dentinogenesis imperfecta, hearing loss, skin hyperlaxity and joint hypermobility [14]. Scoliosis is a common feature, and the combination of chest deformities and scoliosis is responsible for respiratory disorders, which may also lead to death [14]. Although types I and IV are milder and less easily recognised, they should be considered in the differential diagnosis of children with multiple fractures [9,21].

Radiographs commonly reveal cortical bone thinning and excessive trabecular bone transparency, although this finding is subjective and difficult to assess with conventional radiography unless there is a significant reduction in calcified bone mass (approximately 30 to 50%) [22,23]. Bone fragility is also exacerbated by muscle wasting and immobilisation. Nevertheless, even if none of the features are specific, their association, together with a suggestive clinical history (i.e. a propensity for fractures, family history of pre-senile loss of hearing) may suffice to confirm OI diagnosis [22].

Management of OI patients is multidisciplinary and includes specialists in medical OI management, orthopaedics, rehabilitation medicine, paediatric dentistry, and otology/otolaryngology [16]. Mainstays of treatment include limb braces, orthotics to stabilise lax joints, promotion of appropriate physical activity, muscle strengthening, pain management, and physical and occupational therapy to maximise bone stability, improve mobility, prevent contractures, and prevent head and spinal deformity [16]. Some data suggest that the susceptibility to fractures in OI arises from low bone mass due to low bone volume [24-26] or from alterations in the material properties of bone [27]. Histomorphometric studies conducted on bone biopsy samples from children with OI types I, III and IV revealed that bone acquisition during growth is profoundly disturbed due to abnormal bone modelling and decreased production of secondary trabeculae during endochondral ossification, as well as decreased thickening of existing trabeculae by bone remodelling [24]. In these patients, early high-resolution transmission electron microscopy observations of bone fragments revealed overmineralised regions, with generally small and unorganised apatite crystals [28]. The bones from OI patients have a greater mineral content but not a larger particle width, which in combination with the decreased BMD might contribute to compromised bone strength [29].

In most OI patients, however, BMD is below the normal range [30], which is the most important risk factor for further fractures [30]. Commonly, children with OI are prone to relative bone loss during growth, with impairment of bone accrual and peak bone mass [31,32]. To date, however, only a small number of longitudinal studies of OI on BMD during growth have been published [32-37]. In particular, in 9-year follow-ups, OI patients appeared to have increased BMD [37].

The use of other techniques for evaluating bone mass and quality in OI has led to similar results. For example, peripheral quantitative computed tomography (pQCT) data suggest that adults with type I OI have an altered bone geometry and microstructure (lower radius total bone area, decreased trabecular number, increased trabecular spacing, and greater trabecular inhomogeneity) and lower bone mass (decreased areal and volumetric BMD) compared to healthy controls. These results suggest that the increased risk of fractures in patients with type I OI may be a combined result of altered bone matrix quality, low bone mass, and altered bone microstructure and geometry [38]. On the other hand, children with OI have low bone quantitative ultrasonography (QUS) values, even if there is no relationship with the number of fractures [39].

Serum calcium is normal in OI subjects, but hypercalciuria has been reported in some patients in the absence of immobilisation, renal dysfunction or nephrocalcinosis. Serum 25OH vitamin D [25(OH)D] levels are often low, indicating a low exposure to sunlight, but serum 25(OH)D levels appear to be positively associated with aBMD (areal BMD) z-scores in children and adolescents with OI types I, III, and IV [40]. Thus, the management of osteoporosis in OI is important and should focus on altering bone resorption, the most detrimental part of bone disease [41].

Although no curative therapy is available for this rare disease, various pharmacological substances have been tested as treatments. Over the last two decades, intravenous bisphosphonates for treatment of patients with OI have shown promising results [42]. Poyrazoglu et al. treated 35 paediatric OI patients with pamidronate. The treatment was associated with increased BMD scores, decreased bone turnover as assessed by bone turnover markers, and lower fracture rates. It was also found that earlier age at the onset of therapy was related to a better response and higher BMD values [42]. Treatment with cyclic intravenous pamidronate in children younger than 3 years old with moderate to severe OI was well tolerated and associated with increased vertebral BMD and reduced fracture frequency [43,44]. The impact of at least 2 years of pamidronate treatment in children with OI revealed an important improvement in cortical width, trabecular bone volume and trabecular number [45]. Human growth hormone therapy as an adjunct to neridronate treatment was also correlated with improved linear growth and increased BMD [46].

Rauch et al. [47] conducted a double-blind placebo-controlled study with oral risedronate for mild OI for two years, providing lower serum bone resorption marker levels and significantly higher aBMD values compared to controls. Bishop et al. reported similar findings in a randomised, double-blind, placebo-controlled trial [10]. Finally, in a randomised 2-year study of oral olpadronate in children with OI, a 31% reduction in fracture risk was observed [48].

In another study, Pediatric Outcomes Data Collection Instrument (PODCI) scores in the sports/physical functioning domain were significantly improved after pamidronate therapy [49], and motor milestones were reached at an earlier age [43]. In OI, physical activity has several advantages. It provides gravitational stressors required for bone growth and remodelling, the muscles supporting joints are strengthened by activity, and as an overall benefit, joint stability is improved [23].

#### ***Bruck syndrome***

Bruck syndrome (BRKS) is a recessive disorder that was first described by Bruck in 1897 [50] and is characterised by congenital contractures and bone fragility. Bruck syndrome type 2 (BRKS2*; OMIM #609220*) is caused by mutations in *Procollagen-Lysine, 2-Oxoglutarate 5-Dioxygenase 2* (*PLOD2*; OMIM *601865), encoding collagen lysyl hydroxylase on chromosome 3q23-q24, whereas Bruck syndrome type 1 (BRKS*1; OMIM #259450*) has been mapped to chromosome 17p12. This syndrome may be caused by a homozygous mutation in the *FK506-Binding Protein 10* gene (*FKBP10*; OMIM *607063) on chromosome 17q21 [51]. These data suggest that a set of genes, such as *FKBP10* and *PLOD2*, act during procollagen maturation to contribute to molecular stability and post-translational modification of type I procollagen, without which bone mass and quality are abnormal [51]. *PLOD2* is a member of the PLOD family of proteins responsible for lysyl hydroxylation. *PLOD* mutations cause Ehlers-Danlos syndrome as well as BRKS type 2 [52]. In contrast, *FKBP10* is a member of the immunophilins, a family of proteins with PPIase activity, which leads to the proper folding of type I collagen prior to assembly of the triple helices [53].

Clinically, most BRKS patients present with congenital contractures and pterygia, with white sclera and normal hearing and vision [54]. The disease progresses relentlessly in all patients and leads to severe limb deformities, short stature, progressive kyphoscoliosis and multiple fractures [55]. Bone fractures occur postnatally, whereas contractures are a primary abnormality and not a complication of fractures [54,55]. Brenner et al. used electron microscopy to examine a bone specimen from an affected patient and observed osteoblasts with swollen mitochondria and dilated endoplasmic reticula, as well as a decrease in the diameter of the collagen fibrils along with a low mineral content and increased pepsin extraction of collagen 1 [56].

The data on bone metabolism, density or quality in BRKS are poor. Plasma calcium and phosphate concentrations appear to be normal in regard to bone formation markers alkaline phosphatase and procollagen-1 N-propeptide [51]. BMD z-scores are in the normal range, even if DXA scanning at the spine and femoral sites often produces artefacts from scoliosis, fractures and acetabular protrusion [51]. Transiliac bone biopsy results reveal trabecular osteopenia and cortical width reduction, without mineralisation defects [51]. Under polarised light, the bone has a normal lamellar structure [51]. While orthopaedic care with fracture management and rehabilitation remain the cornerstones in the management of all types of OI, bisphosphonates have been shown to be safe and effective in the treatment of osteoporosis in BRKS and are now the gold standard of treatment for this syndrome [57].

#### ***Idiopathic juvenile osteoporosis***

Idiopathic juvenile osteoporosis (IJO; *OMIM 259750*) is a rare disorder recognised by Dent and Friedman in 1965 [58]. It is characterised clinically by an insidious onset of bone pain, followed by vertebral compression and repeated fractures of the long bones [59]. The exact prevalence is unknown, but the estimated incidence is 1 in 100,000 [9]. IJO is usually a self-limiting disease, with prepubertal onset, typically between 8 and 12 years of age, and spontaneously resolves after puberty, even if it may result in severe deformities and functional impairment [59].

Two to three years before the onset of puberty, children characteristically experience an insidious onset of pain in the back and lower extremities in conjunction with vertebral compression fractures, difficulty in walking, and multiple fractures of the long bones, especially around the weight-bearing joints [60], without family history of childhood bone diseases, extra-skeletal manifestations, or growth impairment [60,61]. Knee and ankle pain, kyphosis, loss of height and a sunken chest may also be present. Rarely, in more severe cases, permanent disability can develop [61]. The aetiology is unclear, although bone histology indicates reduced bone formation, with a decrease in trabecular bone volume, thickness and number, but without any alterations in cortical bone [60,62]. Diagnosis is based on clinical presentation, skeletal X-rays and bone density tests (DXA, pQCT) [60,61].

Using only DXA [63], or DXA and QUS [64], BMD and bone quality parameters have been reported to be lower than normal for age. Using DXA and bone biopsy to examine a cohort of 24 children, it was reported that histomorphometric findings correlated poorly with fracture history, circulating bone biomarkers and DXA results. However, it was also observed that vitamin D deficiency and reduced BMD were associated with high bone turnover by biopsy [65]. In a recent study using pQCT and bone biopsy, JIO patients were characterised by decreased bone turnover and lower trabecular and cortical BMD Z-scores, with a relationship between both cortical and trabecular bone density and parameters of bone histology [59]. These results support a new method of non-invasive evaluation of cortical and vertebral bone volume [59]. These data suggest that JIO may be due to the presence of a normal number of osteoblasts whose function is altered, leading to a decreased rate of matrix deposition, confirming the decreased bone turnover of the majority of patients, as demonstrated by Rauch et al. [59,62].

The management of JIO is aimed at protecting the spine and other bones from fracture. Physical therapy and exercise (avoiding weight-bearing activities) and other supportive measures are mandatory. There is no established treatment strategy. Treatments with calcium or vitamin D, fluoride, calcitonin, or anabolic steroids have failed to modify the course of IJO, with conflicting data showing improved BMD and fracture rates [65-69].

Bisphosphonates treatment of severe, long-lasting cases have shown to have unequivocal efficacy [60,61,64], even if the spontaneous recovery that may occur during skeletal growth is a confounding factor in examining the effects of treatments. In a recent study, patients with JIO who were treated with bisphosphonates had a complete recovery of painful symptoms, normalisation of bone mineral status at the phalanges of the hand and lumbar spine, and a reduction in fracture rate without changes in linear growth, demonstrating the usefulness of bisphosphonates treatment for this disorder [64].

#### ***Juvenile Paget’s disease***

Juvenile Paget’s disease (JPD; *OMIM #239000*) is a rare autosomal recessive osteopathy characterised biochemically by markedly increased serum alkaline phosphatase (ALP) activity emanating from an increased bone turnover secondary to enhanced osteoclastic activity [70,71]. Approximately 50 cases have been reported worldwide [72]. JPD infants and children typically suffer widespread skeletal involvement manifesting as progressive bone pain, fractures and deformities, short stature, growth retardation, progressive macrocephaly and facial deformity, mainly due to maxillary expansion [72,73]. Compression and trapping of nerves, especially auditory and optic nerves, result in deafness and optic atrophy [74].

Most cases of JPD are caused by osteoprotegerin (OPG) deficiency due to homozygous loss-of-function mutations within the *tumour necrosis factor receptor superfamily member 11b* gene (*TNFRSF11B; OMIM #602643*) that encodes for OPG, located on 8q24.12 [72]. OPG is secreted into the marrow space by preosteoblasts and osteoblasts, acting as a decoy receptor for RANKL to its receptor RANK. Normally, the binding of RANKL to RANK on osteoclast precursors leads to the activation of osteoclastogenesis. By binding to RANKL, OPG reduces its ability to interact with RANK and thus blocks osteoclast formation and bone resorption [72,73]. OPG mutation may therefore lead to uncontrolled osteoclastogenesis.

In JPD patients, X-rays have revealed an undertubulation of long bones with a disorganised trabecular pattern and thin cortices. Rapid rates of skeletal remodelling have been demonstrated by histopathology as well as by biochemical markers of bone turnover [74]. Two other conditions considered in differential diagnosis are polyostotic fibrous dysplasia and hereditary hyperphosphatasia. A markedly elevated alkaline phosphatase level is unusual in polyostotic fibrous dysplasia. Although hereditary hyperphosphatasia is associated with a high level of alkaline phosphatase, it manifests much earlier, and radiographs do not reveal the typical mosaic pattern [72,73]. The morbidity of JPD is very severe, and the majority of children are wheel-chair bound by age 15 if untreated [74]. Recombinant OPG or denosumab, a monoclonal antibody against the receptor activator of nuclear factor-κ ligand (RANKL), are promising therapeutic agents [75], but the data on its use in children are very poor.

Oral bisphosphonate therapy, especially alendronate, importantly suppresses bone turnover and brings about remission [76-79]. Early use in paediatric patients prevents the development of deformities and arrests progression of the disease and further fractures. Although there is concern about permanently disturbing bone remodelling with alendronate, its safe use has been documented in the paediatric age group in recent studies, at least over the short term [80]. Calcitonin is a useful adjuvant, especially for pain relief [81]. In these patients, clinically significant hypocalcaemia seems to be rare after treatment with bisphosphonates, particularly after pamidronate, aledronate, or risedronate treatment [82]. Risk factors for severe bisphosphonate-induced hypocalcaemia include coexisting hypoparathyroidism, vitamin D deficiency, and renal failure [82].

#### ***Early-onset Paget’s disease***

Early-onset Paget’s disease (*OMIM #603499*) of the bone is a very rare genetic disorder that has been reported in only a few families [83,84]. This form, which is inherited in an autosomal dominant pattern, appears commonly in adolescents or young adults. Its features are similar to those of the classic form of the disease, although it is more likely to affect the skull, spine, ribs, and the small bones of the hands [73,83]. Early-onset Paget disease is also associated with hearing loss early in life and defective tooth eruption [85]. People with this disorder have an altered copy of the *tumour necrosis factor receptor superfamily member 11A* gene (*TNFRSF11A; OMIM #603499*), also known as *receptor activator of NF-*κ*e (RANK)*, located at 18q21.33 [73,83].

Radiographs of affected bones show lytic and sclerotic lesions with bony enlargement and deformity. Affected individuals have very elevated serum alkaline phosphatase levels (2 to 17 times the normal range) [85]. The data on treatment options are very sparse. Commonly, surgery has been employed to correct bone deformities. Bisphosphonates have also been used successfully to reduce bone turnover, although the long-term effects of antiresorptive therapy on the natural history of the disease remain unclear [85].

#### ***Hypophosphatasia***

Hypophosphatasia (HPP) is a rare inherited disorder characterised by defective bone and tooth mineralisation and deficiency of the liver/bone/kidney alkaline phosphatase gene [86,87]. The prevalence of severe forms of the disease has been estimated at 1/100,000 [88,89]. Based on clinical course and severity, HP has been divided into 6 major subtypes. The symptoms are highly variable in their clinical expression, ranging from stillbirth without mineralised bone to early loss of teeth without bone symptoms [86-88]. Depending on the age at diagnosis, 6 clinical forms are currently recognised: perinatal (lethal) (*OMIM #241500*), perinatal benign [86-88], infantile (HOPS; *OMIM #241500*), childhood (*OMIM* #241510), adult (*OMIM #146300*), and odontohypophosphatasia (*OMIM #146300*) [86-93]. In lethal HPP, the patients show markedly impaired mineralisation in utero. This form is characterised by stillbirth or death after birth due to hypoplastic lungs and respiratory insufficiency, difficult-to-treat seizures, and hyperkalaemia with extensive hypomineralisation and bone deformities.

Perinatal benign HPP is characterised by prenatal skeletal manifestations that slowly resolve during childhood or adulthood. Infantile HPP has an onset between birth and six months without elevated serum alkaline phosphatase activity, rickets, premature craniosynostosis, irritability, seizures or nephrocalcinosis due to hypercalciuria [89-91]. Childhood hypophosphatasia is characterised by low bone mineral density for age with unexplained fractures or rickets, causing short stature, delayed walking and pain of the lower extremities. Premature loss of teeth often leads to diagnosis [86-91]. Adult HPP presents with osteomalacia, chondrocalcinosis, osteo-arthropathy and stress fractures during middle age [92]. Odontohypophosphatasia has been characterised by the premature exfoliation of primary teeth or severe dental caries, often not associated with abnormalities of the skeletal system [93].

HPP is an inborn error of metabolism characterised biochemically by low serum alkaline phosphatase (ALP) activity (hypophosphatemia) and caused by a loss-of-function mutation within the gene encoding *tissue-nonspecific alkaline phosphatase* (*TNAP*) (ALPL; *OMIM#171760*) on chromosome 1p36 [86-88]. Consequently, natural substrates of this cell surface enzyme, such as phosphoethanolamine (PEA), pyridoxal 5′phosphate (PLP), and inorganic pyrophosphate (PPi), of this cell surface enzyme accumulate extracellularly [85], blocking hydroxyapatite crystal propagation and thereby causing rickets during growth or osteomalacia in adults [86]. There is no curative treatment for hypophosphatasia, but symptomatic treatments such as non-steroidal anti-inflammatory drugs or teriparatide have been shown to be beneficial [94-96]. Enzyme replacement therapy will certainly be the most promising challenge of the next few years [97].

#### ***X-linked hypophosphatemic rickets***

X-linked hypophosphatemic (XLH; *OMIM #307800*) rickets is a sex-linked dominant disorder of phosphate homeostasis characterised by defective renal phosphate handling and vitamin D metabolism, which leads to growth retardation, rachitic and osteomalacic bone disease, and hypophosphatemia [98]. Albright first described XLH in 1939, and it is the most common form of inherited rickets, with an incidence of 1 in 20,000 [98]. XLH accounts for more than 80% of familial hypophosphatemic rickets [99].

Inactivating mutations of the *Phosphate-regulating gene with Homologies to Endopeptidase on X chromosome* (*PHEX*; OMIM *300550) are responsible for the XLH phenotype [100-102]. *PHEX* encodes a metalloprotease that cleaves small peptide hormones and is expressed in the bones, teeth, and parathyroid glands, but not in the kidney. Fibroblast growth factor 23 (FGF23) is the major phosphatonin, and increased levels have been observed in XLH [99]. It does not seem to cleave FGF23 directly, but it is involved in the down-regulation of FGF23 by an unknown mechanism [100]. Such mutations are speculated to increase the levels of phosphatonins by reduced degradation or increased production [101,103].

Phosphatonins are circulating factors primarily produced in bone that act on proximal renal tubular cells to increase phosphate wasting by the down-regulation of sodium-phosphate co-transporters [101]. In addition, they reduce the activity of the 1a-hydroxylase enzyme, leading to inappropriate levels of 1,25 (OH)2 vitamin D3 in the face of hypophosphatemia [101]. Mutations can be detected in 50-70% of the affected patients. In addition, the severity of the disease and specific clinical manifestations are variable even among members of the same family. In a recent study, patients with clearly deleterious *PHEX* mutations that resulted in premature stop codons had lower tubular reabsorption of phosphate and 1,25(OH)_2_D levels than those with plausible causative mutations [103-105].

Untreated XLH is associated with growth retardation and bone deformities, whereas treatment with oral phosphate and vitamin D preparations may improve growth [104] but is associated with complications, including nephrocalcinosis, hyperparathyroidism, hypertension, and cardiovascular abnormalities. Hyperparathyroidism may be an early event in the development of other complications, particularly hypertension. Measures to prevent hyperparathyroidism include the appropriate dosing of phosphate and vitamin D [104]. In addition to the mineralisation defect induced by hypophosphatemia, an intrinsic osteoblast defect also contributes to the bone disease and does not appear to respond to conventional treatment [105].

Children and adolescents who are being treated manifest a bone mineral disorder characterised by decreased BMD in the appendicular skeleton and increased BMD in the lumbar spine [106]. This outcome has also been observed in adults during DXA examination of the lumbar spine [107]. Bone mineral content was significantly higher in the XLH cohort in a pQCT study conducted in children, adolescents and adults with XLH [108]. In pQCT, XLH patients appear to have elevated trabecular volumetric bone mineral density (vBMD) at the distal radius while receiving calcitriol and phosphate supplementation but low cortical vBMD at the radial diaphysis. Low cortical vBMD presumably reflects the underlying mineralisation defect that is not entirely corrected by current treatment approaches [108]. However, XLH patients have a muscle function deficit in the lower extremities, even if bone mass and size are increased at the distal tibia [109].

Conventional combined treatment of 1,25 dihydroxyvitamin D3 and inorganic phosphate salts has been thoroughly demonstrated to improve linear growth and heal rachitic skeletal abnormalities. However, severe growth retardation is common in some patients, even with early medical intervention [98,99]. Moreover, the calcimimetic drug cinacalcet has been reported to be effective in XLH [110].

#### ***Hypophosphataemic nephrolithiasis/osteoporosis***

Hypophosphataemic nephrolithiasis/osteoporosis-1 (NPHLOP1; *OMIM #612286*) is caused by a heterozygous mutation in the *Solute Carrier Family 34 (Sodium/Phosphate Cotransporter) Member 1* gene (*SLC34A1*; *182309) on chromosome 5q35 [111]. Hypophosphataemic nephrolithiasis/osteoporosis-2 (NPHLOP2; *OMIM #612287*) is caused by a heterozygous mutation in the *Solute Carrier Family 9, Member 3, Regulator 1* gene (*SLC9A3R1*; OMIM *604990) on chromosome 17q25.1 [112]. Among 20 patients with urolithiasis or osteoporosis and persistent idiopathic hypophosphataemia associated with a decrease in maximal renal phosphate resorption, two were found to have mutations in the *neomycin phosphotransferase 2* (*NPT2*) gene [113].

#### ***Wilson disease***

Wilson disease (WD; *OMIM #277900*) is a rare autosomal recessive disorder with an estimated prevalence rate of 30 cases per million (i.e. one per 30,000) and an incidence of one case per 30,000 to 40,000 births [9]. In the United States, there are approximately 600 cases of WD, and it has been estimated that 1% of the population are carriers [113]. A mutation in the *ATPase, Cu(2+)-Transporting, Beta Polypeptide* gene (*ATP7B*; OMIM *606882) located on chromosome 13q14.3 is responsible for WD [114-116].

Bone and joint involvement are under-recognised components of WD, with radiographic evidence of osteoporosis present in up to 88% of persons with the disease [117-119] that may result in spontaneous fractures. Joint involvement, particularly of the knees, is also common, and joint pain has been reported as the presenting symptom of WD [119]. Radiological evidence of vertebral column abnormalities is evident in 20 to 33% of individuals with WD [117,120].

#### ***Menkes’ kinky hair syndrome***

Menkes’ kinky hair syndrome (MKD; *OMIM #309400*) is a severe multisystem disorder that is caused by defective bioavailability and transport of copper at the cellular level. MKD is caused by a mutation in the gene encoding *Cu(2+)-transporting ATPase, alpha polypeptide* (*ATP7A*; OMIM *300011) located in Xq21.1 [121,122]. MKD is a rare X-linked recessive disorder with an incidence of approximately 1:298,000 [122]. Classical MKD is characterised by mental retardation, hypothermia, seizures, cutis laxa, hypo-pigmentation, abnormal hair (kinky hair, or *pili torti*), and decreased serum ceruloplasmin levels [121,122].

In these patients, the deficient activity of lysyl oxidase, a copper-dependent enzyme, causes defective collagen cross-linking that leads to osteoporosis and pathological fractures. In a study evaluating the changes in BMD following pamidronate treatment in children with MKD, increases in lumbar spine BMC and aBMD were reported as 34-55% and 16-36%, respectively, following 1 year of treatment with pamidronate [123]. No further fractures occurred in two of the three children treated, and no adverse effects of pamidronate treatment were noted [123].

#### ***Osteoporosis-pseudoglioma syndrome***

Osteoporosis-pseudoglioma syndrome (OPPG; *OMIM #259770*) is a rare autosomal recessive syndrome, with only approximately 60 cases reported in the literature [124]. OPPG is characterised by the early onset of severe symptomatic osteoporosis and the loss of vision in infancy [125-130]. The vision-loss phenotype is secondary to defective vascularisation. Most patients are congenitally blind or become blind in early childhood, and all are blind by the age of 25 years [126,128,131]. Mental development is usually normal, but approximately 25% of patients have cognitive impairment [126].

The phenotype is variable, even among siblings [128]. Carriers of the heterozygous mutation also exhibit an osteoporotic bone phenotype [132-134]. In OPPG patients, osteoporosis usually manifests in early childhood with vertebral compression fractures, long bone recurrent fractures resulting from minimal trauma, and reduced BMD. More severely affected patients may show muscle weakness, bowing of the long bones, and severe spinal deformities [128,130,135].

In 2001, mutations in the gene encoding *low-density lipoprotein receptor-related protein 5* (*LRP5*; OMIM *603506) were detected [126,127]. Numerous loss-of-function mutations have been shown to cause OPPG in individuals with homozygous or compound heterozygous mutations [126,136-139]. Additionally, mutations in *LRP5* have been linked to the recessive form of familial exudative vitreoretinopathy (FEVR; *OMIM #601813*) [140]. In contrast, gain-of-function mutations in *LRP5* may underlie autosomal dominant disorders with increased bone mass [141]. The LRP5 expressed in many tissues [142] functions as a transmembrane coreceptor in the canonical Wnt (wingless) signalling pathway, which regulates the growth and differentiation of osteoblasts [127,136-141,143-147]. Moreover, LRP5 may function indirectly by inhibiting the expression of Tph1, a rate-limiting enzyme for serotonin in the duodenum, resulting in higher blood serotonin levels with inhibited osteoblast proliferation and reduced bone formation [142]. Of the approximately 60 previously published *LRP5* mutations associated with OPPG or FEVR, only a few are splice-site mutations [146,148].

Many heterozygous carriers who underwent bone densitometry were found to have low BMD and diagnoses of both osteoporosis and osteopenia. This finding is in accordance with previously published data demonstrating that heterozygous *LRP5* mutations have a predominantly negative effect on BMD [131,145]. In addition, recent studies on *LRP5* polymorphisms and genome-wide association studies have revealed that common sequence variants in *LRP5* influence BMD in the general population [149-152].

Streeten et al. [139] reported nine new cases of OPPG. Of these, four patients were receiving bisphosphonates regularly with good responses. Barros et al. [153] reported similar findings in two brothers with OPPG who were treated for 4 years and exhibited decreased fracture events. As a result, the higher BMD was cited as clinically significant. A marked increase in BMD of the lumbar spine and femur hip was reported in a study that involved the administration of teriparatide for 2 years following a 6-year course of intravenous pamidronate infusions [154]. While an increase in CTX followed by an increase in P1NP is an unusual sequence of events when teriparatide is used to treat osteoporosis, this finding may be representative of low bone turnover states [154].

#### ***Homocystinuria***

Severe hyperhomocysteinaemia (HHcy, total plasma levels of homocysteine >50 μM) or homocystinuria (*OMIM #236200*) can be caused by defects in remethylation or transsulphuration. Disturbed remethylation due to a deficiency of *5,10-Methylenetetrahydrofolate Reductase* (*MTHFR*; OMIM *607093) as well as *5-Methyltetrahydrofolate-Homocysteine S-Methyltransferase* (*MTR*; OMIM *156570) [155] result in elevated homocysteine and decreased methionine. In the transsulphuration pathway, CBS deficiency also results in an accumulation of homocysteine, and in contrast to remethylation defects, methionine is increased [156].

Highly variable neurological presentations that range from schizophrenia and depression to severe mental retardation are common in severe hyperhomocysteinemia, independently of arterial and venous occlusive disease and regardless of defects in remethylation or transsulphuration [157]. The increased prevalence of osteoporosis in patients with homocystinuria suggests that a high serum homocysteine concentration may weaken bone by interfering with collagen cross linking, thereby increasing the risk of fracture [156]. These findings suggest that the homocysteine concentration, which is easily modifiable by dietary intervention, is an important risk factor for hip fractures in older people [158,159].

Homocysteine (Hcy) modulates this process via several mechanisms, such as by increasing osteoclast activity, decreasing osteoblast activity, and by direct Hcy effects on bone matrix. A detrimental effect on bone is also possible due to a decrease in bone blood flow and an increase in matrix metalloproteinases (MMPs) that degrade the extracellular bone matrix. Notably, Hcy has been demonstrated to bind directly to the extracellular matrix and to reduce bone strength [158,159]. With regard to whether Hcy affects bone density, earlier studies reported an alteration in bone biomechanical properties in conditions associated with deficiencies of vitamin B12, folate, and HHcy. Moreover, existing data leave open to speculation whether folate and vitamin therapy also acts via Hcy-independent pathways [158,159].

Several bone markers may be used to examine the effects of high levels of plasma Hcy (hyperhomocysteinemia) on bone, i.e. hydroxyproline and N-terminal collagen 1 telopeptides. In HHcy, mitochondrial abnormalities have been identified. The mechanism of Hcy-induced bone remodelling via the mitochondrial pathway is largely unknown [158,159]. Some studies have demonstrated that the administration of homocysteine induces osteopenia in new-born rats [160]. In addition, these data suggest that hyperhomocysteinemia may disrupt the normal development of epiphyseal cartilage in the rat embryo [160]. However, whereas most homocystinuria patients diagnosed in adulthood have severe osteoporosis, a good control over plasma homocysteine appears to prevent or delay some of these complications [161]. However, more studies are needed to clarify the mechanistic role of Hcy in bone diseases.

#### ***EhlersnDanlos syndrome***

Ehlers-Danlos syndrome (EDS) is an inherited disorder of connective tissue characterised by hyperextensible skin, joint laxity, and easy bruising [162,163]. In the more common EDS types I, II, and III, the underlying genetic defect is unclear, but a recent study has suggested a linkage with the *COL5A1* gene [162]. The more rare type IV EDS, in which vascular fragility predominates, results from a deficiency of type III collagen [163,164] (Table 3).

**Table 3:** Causes of Ehlers-Danlos syndrome, genes involved, location, inheritance, and gene product

**Ehlers-Danlos syndrome**	**OMIM**	**Inheritance**	**Gene**	**Location**	**Gene product**
Type I	130000	AD	*COL5A2*	2q32.2	Collagen, Type -V, Alpha-2
	130000	AD	*COL5A1*	9q34.3	Collagen, Type -V, Alpha-1
	130000	AD	*COL1A1*	17q21.33	Collagen, Type -I, Alpha-1
Type II	130010	AD	*COL5A1*	9q34.3	Collagen, Type -V, Alpha-1
Type III	130020	AD	*TNXB*	6p21.33	Tenascin XB
Type IV	130050	AD	*COL3A1*	2q32.2	Collagen, Type III, Alpha-1
Type V	305200	-	-	-	-
Type VI	225400	Ad/ar	*PLOD1*	1p36.22	Procollagen-Lysine, 2-Oxoglutarate 5-Dioxygenase
Type VIIA	130060	AD	*COL1A1*	17q21.33	Collagen, Type I, Alpha-1
Type VIIB	130060	AD	*COL1A2*	7q21.3	Collagen, Type I, Alpha-2
Type VIIC	225410	AR	*ADANTS2*	5q35.2	Procollagen I N-Proteinase
Type VIII	130080	AR	-	12p13	-

Note: This table lists only the most frequent types according to the recent literature.

Reduced bone mass does not appear to be one of the cardinal features of EDS and has not been described in early reports [165,166]. Nevertheless, some have reported that nearly 100% of EDS patients may have osteopenia or osteoporosis [164]. However, osteopenia has been reported to occur in the very rare type VI EDS together with muscle hypotonia, kyphoscoliosis, and ocular globe rupture [162]. Some recent reports have suggested that bone density may be reduced in EDS [163,166-168], although the small sample sizes and possible referral and selection bias limit the interpretation of these findings. In a case–control study, EDS patients demonstrated differences in fracture rates, bone mass, and heel ultrasound parameters compared with age- and sex-matched controls [166]. Notably, some studies have identified a likelihood of fracture, low bone mass, and abnormal bone structure among EDS patients [162,166]. The aetiology is likely to be multifactorial with the involvement of an inherited structural element and may be exacerbated by immobility or reduced exercise [162].

#### ***Marfan syndrome***

Marfan syndrome (MFS; *OMIM #154700*) is an autosomal dominant connective tissue disorder with variable expressivity and skeletal, cardiovascular, and ocular involvement. The prevalence is approximately 1 per 10,000 subjects [169]. MFS is caused by mutations in the *Fibrillin 1* gene (*FBN1*; OMIM *134797) [170-172] that encodes for a glycoprotein that aggregates to form microfibrils.

*FBN1* haploinsufficiency is a major pathogenetic mechanism in MFS [173]. Earlier theories proposed that abnormal microfibrils altered the entire fibril structure (with a predominantly negative effect), thereby resulting in the disease phenotype. However, certain features in MFS, such as long bone and rib overgrowth as well as muscle wasting, cannot be explained by this phenomenon. The loss of fibrillin 1 protein has a subsequent effect on the pool of TGF-β, a factor that plays a critical role in bone health, thus giving rise to various phenotypic manifestations of the disease [174]. This effect could be modulated further by different variants of FBN-1, which may explain the variability in clinical presentation [173,175].

Notably, TGF-β has been demonstrated to positively regulate osteoblast proliferation and differentiation in vitro [176], with *TGF-β*^*-/-*^ mice exhibiting low bone mass and poor bone quality [177]. In addition, TGF-β released during bone resorption induces the migration of bone mesenchymal stem cells, which differentiate further into osteoblasts. However, the over-expression of TGF-β from osteoblasts leads to low bone mass due to the stimulation of osteoclastogenesis [177]. TGF-β antibody has also been demonstrated to increase BMD, trabecular thickness, and bone volume by increasing the number of osteoblasts and reducing the number of osteoclasts [178]. Moreover, Fbn1mgR/mgR mice (a model of severe MFS) exhibited decreased bone volume and density due to TGF-β driven osteoclastogenesis [179].

Reduced axial and peripheral BMD have been observed in several studies in adults with MFS, suggesting that these patients are at an increased risk of fractures [180-187]. Conversely, evidence of osteopenia has not been encountered in some cases [188]. Nonetheless, there is a paucity of data regarding the bone mineral status of MFS patients during childhood. In a study involving 16 MFS patients, a low BMD at the femoral neck and a trend towards reduced lumbar spine BMD were reported [189]. Other data indicated that MFS children have significantly lower BMC as well as whole-body and lumbar-spine BMD compared with controls that were matched for age, sex, height, and ethnicity [189]. In contrast, another study reported normal BMD at the lumbar spine and femoral neck in 21 MFS children [186].

#### ***Hajdu-Cheney Syndrome***

Hajdu-Cheney syndrome (HJCYS; *OMIM #102500*) is a rare autosomal dominant skeletal disorder that was first reported by Hajdu and Kauntze [190] and later by Cheney [191]. It is caused by heterozygous activating mutations in the *NOTCH homolog 2 (Drosophila)* gene (*NOTCH2*; OMIM *600275) on chromosome 1p13-p11. Approximately 60 cases of HJCYS have been reported [192], and nearly 24 mutations of *NOTCH2* have been identified, with 12 of these representing missense/nonsense mutations [193].

Notch signalling typically triggers the segmentation of the axial skeleton during somitogenesis [194] and controls bone remodelling, particularly suppressing osteoblast maturation and function, and also bone resorption by inducing osteoprotegerin expression in osteoblasts [194]. HJCYS is characterised by short stature, coarse and dysmorphic facies, bowing of the long bones, vertebral anomalies, and decreased bone density. Facial features include hypertelorism, bushy eyebrows, micrognathia, small mouth with dental anomalies, low-set ears, and a short neck. These patients also have wormian bones, open sutures of the skull, and platybasia. Additional and variable features include joint hypermobility, hearing loss, renal cysts, sellar and clivus abnormalities (such as an enlarged sella turcica), and cardiovascular anomalies [195-199].

Progressive focal bone destruction occurs in HJCYS patients and includes acroosteolysis and severe generalised osteoporosis [198]. Precocious osteoporosis may also develop in these patients [200], and marked decreases in BMD are evident particularly in the spine as compared to age-adjusted normal range values [192]. No definitive studies have indicated the mechanisms responsible for generalised osteoporosis. Focal osteolysis is accompanied by neovascularisation, inflammation, and fibrosis [195,201,202]. Iliac crest biopsies, as reported in a small number of HJCYS cases, revealed decreased trabecular bone, normal or increased bone remodelling, and normal or decreased bone formation [203-206]. Some studies have reported that osteoclast activity may be increased [201,203,204], normal [205,206], or decreased [202] in HJCYS. However, a reduced rate of bone formation has also been described [201,203,205].

In these patients, osteoporosis treatment outcomes have rarely been reported [207-209]. Bisphosphonate therapy has been attempted alone or in combination with teriparatide, but there is no clear evidence regarding the actual benefit of these therapies [204,207,210]. Some have demonstrated a good response with an increase in BMD following teriparatide therapy [208], whereas others have demonstrated favourable effects of zoledronic acid therapy [209].

#### ***Torg-Winchester syndrome***

Torg-Winchester syndrome (TWS; *OMIM #259600*) is caused by a mutation in the gene encoding *matrix metalloproteinase-2* (*MMP2*; OMIM *120360) located in 16q12.2 [210]. The matrix metalloproteinases are a group of enzymes involved in collagen homeostasis. The exact mechanism explaining the progression to osteolysis is unknown, but it is believed that abnormal collagen breakdown may lead to abnormal osteoblast activity with subsequent osteolysis [211,212].

TWS is an autosomal recessive multicentric osteolysis with predominant involvement of the hands and feet. In the past, TWS was reported as 3 separate entities: Torg syndrome, Winchester syndrome, and NAO syndrome. The 2006 revision of the Nosology of Constitutional Disorders of Bone classified Torg and Winchester syndromes as a single entity with NAO syndrome as a variant [213].

Zankl et al. defined the continuous clinical spectrum of Torg-Winchester syndrome [212]. Torg syndrome is characterised by the presence of multiple, painless, subcutaneous nodules, as well as mild to moderate osteoporosis, and osteolysis that is usually limited to the hands and feet. Radiographically, osteolysis is accompanied by a characteristic widening of the metacarpal and metatarsal bones. Winchester syndrome presents with severe osteolysis in the hands and feet with generalised osteoporosis and bone thinning, similar to NAO, but subcutaneous nodules are characteristically absent. Various additional features include coarse facies, corneal opacities, gum hypertrophy, and EKG changes. NAO syndrome, which has only been described in patients from Saudi Arabia, is generally more severe with multiple prominent and painful subcutaneous nodules, massive osteolysis in the hands and feet, and generalised osteoporosis. Coarse facies and body hirsutism are additional features [214-217].

Mosig et al. generated *Mmp2-/-* mice and observed attenuated features of human multicentric osteolysis with arthritis, including progressive loss of bone mineral density, articular cartilage destruction, and abnormal long bone and craniofacial development. These changes were associated with marked and developmentally restricted decreases in osteoblast and osteoclast numbers in vivo. *Mmp2-/-* mice had approximately 50% fewer osteoblasts and osteoclasts than control littermates at 4 days of life, but these differences were nearly resolved by 4 weeks of age [218]. Two siblings affected by Torg-Winchester syndrome were administered intravenous pamidronate over a period of 3 years with no evidence of clinical improvement. Although the bone mineral density of the axial skeleton appeared improved, osteoporosis and osteolysis of the appendicular skeleton continued to worsen [219].

#### ***Shwachman-Diamond syndrome***

Shwachman-Diamond syndrome (SDS; *OMIM #260400*) is a rare autosomal recessive disorder characterised by exocrine pancreatic insufficiency, bone marrow failure with varying degrees of hypoplasia and fat infiltration, and skeletal abnormalities [220]. SDS results from mutations in the ubiquitously expressed and conserved *Shwachman-Bodian-Diamond syndrome* gene (*SBDS*; OMIM *607444) [221]. However, SDS defects are relatively organ specific (i.e. the pancreas, liver, bone marrow, and bone) [222].

SDS patients frequently present with failure to thrive, susceptibility to infections, and short stature. A persistent or intermittent neutropenia occurs in 88-100% of patients. Many patients may exhibit skeletal abnormalities, such as delayed secondary ossification, abnormal development of growth plates and metaphyses, progressive thinning and irregularity of growth cartilage with possible asymmetric growth, and generalised osteopenia and osteoporosis [220,223].

In addition to the skeletal dysplasia, SDS is associated with low bone mass, low bone turnover, and vertebral fragility fractures [224]. Osteoporosis may result from a primary defect in bone metabolism and may be related to bone marrow dysfunction and neutropenia [224]. Of note, *Sbds* is required for in vitro and in vivo osteoclastogenesis (OCG) [224]. Sbds-null murine monocytes formed osteoclasts of reduced number and size due to impaired migration and fusion, which are two functions required for OCG [224]. Phenotypically, Sbds-null mice exhibited low-turnover osteoporosis, which was consistent with findings observed in SDS patients [222]. This osteoporosis was attributed to impaired signalling downstream of the receptor activator of nuclear factor-_k_B (RANK) and to reduced expression of the RANK-ligand-dependent fusion receptor DC-STAMP [222].

In constitutive and inducible SBDS-depleted HeLa cell clones, a 3- to 6-fold elevation was observed in mRNA levels of osteoprotegerin (OPG or TNFRSF11B) and vascular endothelial growth factor-A (VEGF-A) [225]. Osteoprotegerin and VEGF-A are known to have diverse effects on osteoclast differentiation, angiogenesis, and monocyte/macrophage migration, all of which are processes that may be aberrant in SDS, and we propose that overexpression of these factors may contribute to the pathology [224].

Bone biopsies in some SDS patients exhibited significant low-turnover osteoporosis with reduced trabecular bone volume, low numbers of osteoclasts and osteoblasts, and a reduced amount of osteoids [224]. In SDS patients, the main findings were as follows: (1) markedly reduced BMD Z-scores at the lumbar spine (median -2.1, range -4.4 to -0.8) and proximal femur (median -1.3, range -2.2 to -0.7) and reduced Z-scores for height-adjusted BMC/LTM ratio (median -0.9, range -3.6 to +1.1) [223]; (2) vertebral compression fractures in some patients [120]; and (3) blood biochemistry suggestive of mild vitamin D and vitamin K deficiency [224].

## Discussion

Awareness and recognition of the syndromes and genetic disorders associated with a high risk of osteoporosis in childhood carry particular relevance in light of recent research, which has shown that primary osteoporosis does not truly represent a rare condition [1,226,227]. Moreover, as paediatricians may not recognise the risk for bone loss in children, treatment for such bone loss may not be provided. In severe cases of bone loss, as in osteoporosis, a child may develop fractures or symptoms [1,227]. In contrast, in cases of less severe but more chronic forms of bone loss, a child may not attain his or her genetically determined PBM [4]. Such children may be at greater risk for adult-onset osteoporosis since they will enter adulthood with a lower bone mass than would otherwise be anticipated [4].

While the main factors responsible for the achievement of peak bone mass in any healthy individual appear to be genetically determined [228], in children with conditions of reduced mobility who are able to stand, there is evidence that an increased duration of standing or physical activity will improve BMD of the spine and femur [229]. The influence of exercise-induced loading is further dependent on the stage of skeletal maturity, with pre- and early puberty representing the development intervals during which the skeletal response to loading is optimised [230]. In addition, Bowden et al. [231] reported decreased levels of 25-hydroxyvitamin D among a large group of paediatric patients with osteoporosis and osteopenia. Although no direct correlation with fracture risk was detected, it was surmised that vitamin D supplementation in children with osteopenia and osteoporosis is advisable and may indeed reduce morbidity [5].

The recommended daily consumption of vitamin D may be insufficient for patients undergoing bone-affecting treatments and those who suffer from primary bone disorders [5]. In contrast to adult practice, the current evidence base supporting interventions to potentially prevent osteoporosis in children is limited. While calcium and vitamin D supplementation might appear to comprise an appropriate treatment plan for a child with low BMD, there is no good evidence in paediatric practice to support such an approach, unless there is evidence of vitamin D deficiency or poor dietary calcium intake [5].

We have summarised the bone density, quality, and bone metabolism data of some of the more frequent forms of primary osteoporosis. We have also highlighted the risk factors for poor bone health, discussed diagnostic tools, and identified prevention and treatment options. The main shortcoming highlighted is that for some genetic diseases or syndromes, the available data related to bone metabolism, density, and quality are plentiful. For some diseases, studies have applied new methods such as QUS or pQCT. The application of these methods may shed additional light on the characteristic findings with regard to bone metabolism, structure, density, and quality in many of the diseases discussed and may also illuminate other conditions that are associated with primary bone diseases.

In contrast, we encountered very poor data for many other genetic diseases or syndromes that were often limited to few case reports. Moreover, studies that were more than 10 years old lacked attention to data normalisation, e.g. with regard to the height of patients (many diseases that primarily affect the bone tissue involve the pubertal stage or skeletal maturation), and thus provided data of limited utility [232]. The reference data for the definition of osteoporosis have also changed, which introduced difficulties in making comparisons between different studies [233].

Because the exact pathogenic mechanisms in many forms of PO have not been clearly established, it is difficult to proceed with rational drug discovery to specifically address these mechanisms. In other situations, as in copper dysmetabolism syndromes, it is unclear whether specific therapy or bone-loss prevention strategies have been addressed adequately by the appropriate investigations. Frequently, bisphosphonate treatment represents the first-line therapy for many disorders [1,234]. In many of these conditions, bisphosphonates have also been studied as a potential preventive measure [1,234]. Although many different bisphosphonates varying in potency and method of administration are currently available, most of the studies undertaken in children have utilised intravenous preparations of pamidronate [1]. It is also evident that most of the studies to date have been observational, with relatively few randomised controlled trials [234]. Thus, randomised controlled trials are required for many other genetic syndromes in which bisphosphonates are used.

Notably, other new drugs such as recombinant human parathyroid hormone (rhPTH) may prove useful in the treatment of primary osteoporosis. However, several concerns have arisen regarding the safety of such an agent, particularly in younger patients [235], and results from additional studies are required before rhPTH can be accepted as a treatment for OP in children [235]. This may not occur before other specific treatments such as gene therapy or stem cell transplantation become available for certain genetic bone diseases.

## Conclusions

Primary osteoporosis in the paediatric population is a relatively rare disorder. While paediatric primary osteoporosis is characterised by a low incidence of mortality, it carries a considerable burden of morbidity, particularly due to pain, interference with regular activities, and long-term sequelae. It is the responsibility of the physicians who provide care to paediatric patients to maintain working knowledge of the causes of paediatric bone loss so that they may develop appropriate bone-loss prevention or treatment strategies.

## Competing interests

The authors declare that there are no conflicts of interest that could be perceived as prejudicing the impartiality of the research reported.

## Authors’ contributions

SS, LC. SS, MdM, MLB. All authors read and approved the final manuscript.
